# Benefits Conferred by Peer-Support Nursing Intervention to Pulmonary Function and Quality of Life in Nonsmoking Patients with COPD

**DOI:** 10.1155/2021/7450979

**Published:** 2021-06-15

**Authors:** Xiaoling Yao, Xiaoyu Wang, Jing Yuan, Zhikang Huang, Dan Wu, Hongyan Xu

**Affiliations:** Hunan Provincial People's Hospital, The First Affiliated Hospital of Hunan Normal University, Changsha, China

## Abstract

**Objective:**

Peer support is a concept of substantial significance to health scientists and practitioners today due to its focus shifting from disease treatment to health promotion. Effective incorporation peer relationships in support-enhancing interventions could improve quality care and health outcomes. More and more cases of chronic obstructive pulmonary disease (COPD) have been diagnosed in nonsmokers. In this study, the effects of peer-support nursing intervention on the pulmonary function and quality of life of nonsmoking patients with COPD were investigated.

**Methods:**

A total of 100 COPD nonsmoking patients admitted to our hospital from October 2018 to October 2020 were selected as study subjects. All nonsmoking patients were in accordance with the guidelines of COPD diagnosis and treatment issued by the Respiratory Medicine Branch of Chinese Medical Association, and they were not in the habit of smoking. According to the different interventions, the nonsmoking patients were divided into the control group (*n* = 50) and the observation group (*n* = 50). Among them, nonsmoking patients in the control group received routine care, and nonsmoking patients in the observation group received routine care and peer-support nursing. The difference on the scores of social support, self-management efficacy, healthy lifestyle, and the distance of six-minute walking were to be compared between the two groups before and after the intervention.

**Results:**

There was no significant statistical difference on the general information between the two groups in terms of age, gender, and course of disease (*P* > 0.05). Before intervention, the social support score involving subjective support, objective support, utilization of support, and total score revealed slight difference between the two groups (*P* > 0.05). However, after the intervention, the subjective support, utilization of support, and total score remained statistically different between the two groups (*P* < 0.05), and the objective support showed no significant difference between the two groups (*P* > 0.05). Before intervention, there was no statistical difference in the self-management efficacy scores such as positive attitude, stress reduction, self-decision-making, and total score between the two groups (*P* > 0.05). After the intervention, the two groups indicated statistical difference in the self-management efficacy scores (*P* < 0.05). Before intervention, there was no significant difference between the two groups in the healthy lifestyle score in terms of health responsibility, self-realization, interpersonal support, and stress management (*P* > 0.05), and the abovementioned outcome measures indicated significant difference between the two groups after intervention (*P* < 0.05). There was no statistical difference in six-minute walking distance between the two groups before the intervention (*P* > 0.05), but after the intervention, the observation group revealed a significantly longer distance of six-minute walking compared to the control group (*P* < 0.05).

**Conclusion:**

These data suggest that peer-support nursing intervention can effectively improve pulmonary function and quality of life of nonsmoking patients with COPD.

## 1. Background

Chronic obstructive pulmonary disease (COPD) is featured by persistent respiratory symptoms concomitant with incompletely reversible expiratory airflow limitation [1]. COPD is attributed to more than 3 million deaths worldwide every year [2]. Tobacco smoking is the leading cause, but not the only one [3]; unhealthy diet, exacerbations, and physical inactivity, passive mocking, and environmental exposure to biomass fuel are also important reasons to make COPD a major health-care problem [4]. COPD is a complex and heterogeneous disease that requires intensive treatments including long-acting maintenance bronchodilators, inhaled corticosteroids, and pulmonary rehabilitation [5, 6]. All these treatments could decrease symptoms, reduce exacerbation frequency, and optimize functional performance. Despite progression in improvement of symptoms and prevention of acute exacerbations, few advances have been made to reduce disease progression or affect mortality [7]. Although COPD mainly affects pulmonary function, it is now considered to be a complex multicomponent disease characterized by chronic systemic inflammation and often coexists with other comorbidities, such as cardiovascular disease, lung cancer, osteoporosis, muscle weakness, and cachexia [8]. The importance of social relationships in the treatment of disease and the maintenance of health and wellbeing has drawn the attention of scientists and practitioners across a large number of behavioral science and health disciplines. Peer support depends on the belief that people who have faced, endured, and overcome adversity can offer useful support, encouragement, hope, and possibly mentorship to others facing similar situations [9]. Peers are nonprofessionals who have similar diseases or close familiarity with disease management. Effective peer supports offer key functions including assistance in daily management, social and emotional support, linkage to clinical care, and ongoing availability of support [10]. Peer-support nursing can not only effectively make nonsmoking patients feel positive energy such as respect and trust but also enable nonsmoking patients to get guidance from medical staff in the community, so as to promote the establishment and maintenance of their healthy lifestyle [11]. Although there have been different peer-support models, one-on-one, face-to-face, and group Internet peer-support programs should be given priority when considering ways to offer peer support [12]. Peer-led support groups play an important role in supporting people with chronic diseases [13]. The present study focused on the effects of peer-support nursing intervention on the pulmonary function and quality of life of nonsmoking patients with COPD.

## 2. Methods and Materials

### 2.1. General Information

A total of 100 COPD nonsmoking patients admitted to our hospital from October 2018 to October 2020 were selected. All nonsmoking patients complied with the guidelines of COPD diagnosis and treatment proposed by the Respiratory Medicine Branch of the Chinese Medical Association, and none of them had a smoking habit. The nonsmoking patients were divided into two groups involving the control group (*n* = 50) and the observation group (*n* = 50). The control group was treated with routine care, and the observation group was treated with routine care and peer-support nursing. Those nonsmoking patients should be excluded if involved any of the following criteria: (1) other diseases of the respiratory system, such as lung cancer and tuberculosis; (2) severe cardiovascular diseases, such as unstable angina, heart failure, recent myocardial infarction, frequent atrial or ventricular premature beats, severe pulmonary hypertension, and severe liver and kidney dysfunction; (3) severe cognitive impairment and negative treatment compliance. The study was approved by the Hospital Ethics Committee, and the informed consent was signed by the patient voluntarily.

### 2.2. Selection Criteria of Peer and Nurse

Before the study, the eligible nurses and peers were selected in our hospital following the principle of voluntary. The nurses should be well-educated with bachelor degree or above, with more than 5 years' experience in respiratory department and over 3 years' experience worked as a senior nurse in respiratory department. They were trained in the disease material and data collection methods to unify the research method and reduce the deviation. The four peer supporters were selected from the nonsmoking patients with COPD discharged from our hospital and 2 males and 2 females were involved. The peers should be in stable condition, with strong expression ability and responsibility, and received health education and training. All peers were reassessed by participating in the training of COPD health education.

### 2.3. Intervention Methods

The patients in the observation group received the health education training in the form of group interaction and by means of situational dialogue. The training was performed on Monday afternoon and lasted for 2 h. A total of 10 patients were involved at most. The peers were assigned as group leaders to demonstrate the pulmonary function exercises and share disease rehabilitation knowledge. At least one of the peers was participated in the training. The patients were requested to become a member of peer-support WeChat group and participated in the group communication at 8 pm every Sunday. In the WeChat group, the disease rehabilitation knowledge and psychological counseling were shared by the nurses and peers, and the activity lasted for 1 h at least. The nonsmoking patients who were not involved should be supervised by phone. The patients in the control group accepted the routine nursing intervention, such as health education training, WeChat group communication, and psychological counseling, without the peers' participation.

### 2.4. Outcome Measures

The pulmonary function and quality of life of nonsmoking patients in the two groups were evaluated before intervention and after 3 months of intervention. The life quality was measured by the social support, self-management efficacy, and healthy lifestyle. The social support rating scale, as the evaluation of social support scores, mainly included the subjective support, objective support, and utilization of support. There were 10 items in total, with a total score of 66. The higher score was associated with the higher social support [6]. The health promotion self-care scale, as the evaluation of self-management efficacy, mainly contained positive attitude, self-decision-making, and stress reduction. There were 28 items in total and 5-point Likert scale score was applied to the assessment, including 5 scales of no self-confidence, a little bit confidence, confidence, great self-confidence, and overconfidence. The score was positively relevant with the self-management efficacy. The health promotion lifestyle scale, as the evaluation of healthy lifestyle, mainly included six dimensions of health responsibility, self-realization, interpersonal support, nutrition enhancement, regular exercise, and stress management. They were 48 items in total and 4-point Likert scale score was used in the evaluation, involving 4 scales of never, occasionally, often, and always. The higher the score, the better the lifestyle was. The pulmonary function was assessed by six-minute walking distance.

### 2.5. The Six-Minute Walking Distance Test

The six-minute walking distance test was performed on the basis of the American Thoracic Society Guidelines [14]. The nonsmoking patients with comfortable clothes and shoes were required to walk back and forth as fast as possible on a corridor of 30 m, which was marked at each meter, and a seat was set at each end. The walking distance was recorded when the specified time finished. The following points should be attended: 1. avoid flipper-turn and circular route when walking; 2. the nonsmoking patients should be encouraged verbally during walking test; 3. the test was discontinued when the nonsmoking patients felt uncomfortable such as fatigue, dizziness, angina, and dyspnea. 4. The rescue drugs such as nitroglycerin were prepared before the test. Experts who conducted this trial earlier in the United States divided the walking distance of nonsmoking patients into four grades: grade I < 300 m; grade II is 300–374.9 m; grade III is 375–449.5 m; and grade IV > 450 m. The grade was positively related to the cardiopulmonary function.

### 2.6. Statistical Analysis

SPSS 22.0 statistical software was used to process the data. Measurement data were expressed as mean ± standard deviation and analyzed by f test. The counting data were examined by chi-square test. *P* < 0.05 indicates the difference was statistically significant.

## 3. Result

### 3.1. Baseline Demographic by Group

As listed in Table 1, it was found that there were no significant statistical differences between the two groups in terms of age, gender, and disease course (*P* > 0.05).

### 3.2. Peer-Support Nursing Helped Nonsmoking Patients Gain Higher Score of Social Support

Before the intervention, the values of subjective support, objective support, utilization of support, and total score showed slight difference between the two groups (*P* > 0.05). However, at 3 months after the intervention, the above outcome measures, except for objective support, revealed significantly higher score in the observation group compared to the control group (*P* < 0.05, Table 2).

### 3.3. Peer-Support Nursing Improved Self-Management Efficacy of Nonsmoking Patients

As seen in Table 3, the two groups indicated no significant difference in the aspects of positive attitude, stress reduction, self-decision-making, and total score (*P* > 0.05). But at 3 months after the intervention, these values in the observation group were superior to that in the control group (*P* < 0.05).

### 3.4. The Nonsmoking Patients Receiving Peer-Support Nursing Had Healthier Lifestyle

As listed in Table 4, it was found that before the intervention, there was no significant statistical difference between the two groups in terms of health responsibility, self-realization, interpersonal support, and stress management (*P* > 0.05). But at 3 months after the intervention, the score of healthy lifestyle was significantly higher in the observation group than that in the control group (*P* < 0.05).

### 3.5. Peer-Support Nursing Improved Pulmonary Function of Nonsmoking Patients

Before the intervention, there was no significant statistical difference in the six-minute walking distance between the two groups (*P* < 0.05). But at 3 months after the intervention, the nonsmoking patients in the observation group had a significantly longer walking distance of six minutes compared to the control group (*P* < 0.05, Table 5).

## 4. Discussion

With the increase of population and the improvement of health conditions, chronic diseases related to aging and smoking, such as chronic obstructive pulmonary disease (COPD), will be prevalent [15]. Although smoking is the great contributor to the COPD, the nonsmoking COPD patients attracted our attention to a variety of other factors which were related to the COPD [16]. The external risk factors include passive smoking, dust exposure, harmful smoke, air pollution and combustion gas of biomass fuel, and hereditary factor which might be associated with the increasing of the incidence of COPD [17]. The common symptoms of COPD involve chronic cough, excessive sputum production, and breathlessness [18]. The COPD is contributed to the nonsmoking patients' negative attitude and increasing financial burdens [19].

This study was aimed at finding out if the peer-support nursing could improve life quality and pulmonary function for COPD nonsmoking patients. According to the data in this study, we found that there was no significant statistical difference between the two groups in terms of age, gender, and course of disease. But there was significant difference in the score of social support, self-management efficacy, and healthy lifestyle between the two groups at 3 months after the intervention. Several studies have shown that the social support is beneficial for the development and prognosis of disease such as COPD and cancer [20, 21]. As for the social support score including subjective support, objective support, utilization of support, and total score, this study revealed slight difference between the two groups before the intervention. However, at 3 months after the intervention, the observation group indicated significantly higher score of subjective support, utilization of support, and total score than that in the control group, while there was no significant statistical difference in objective support score between the two groups. A study about stroke patients indicated that the self-management program improved patients' self-efficacy and satisfaction of self-management behaviors [22]. In this analysis, the data of the self-management efficacy score showed no significant statistical difference between the two groups before the intervention. But it was found that there was significant statistical difference between the two groups in terms of positive attitude, stress reduction, self-decision-making, and total score at 3 months after the intervention. After comparing the healthy lifestyle of the two groups, it was found that before the intervention, there was slight difference between the two groups in terms of health responsibility, self-realization, interpersonal support, and stress management. But at 3 months after the intervention, these scores in the observation group revealed much higher compared to the control group. The six-minute walking distance test, as a safer and manageable test compared to other walking tests, can better reflect the activities of daily life [23]. As for the comparison of six-minute walking distance between the two groups, we found that there was no significant statistical difference between the two groups before the intervention. But after the intervention, the observation group had a significantly longer walking distance than that in the control group.

In this study, during the hospitalization of COPD nonsmoking patients, nurses formulated nursing plans according to the patients' condition and guided the nonsmoking patients to carry out rehabilitation exercise so as to establish a good nurse-patient relationship. In peer-support nursing, the peers provided comforts and shared similar experience to the COPD nonsmoking patients, which can effectively correct nonsmoking patients' negative psychology, and helped nonsmoking patients build confidence in long-term treatment. In recent years, peer-support nursing has been widely used in the health management of patients with chronic diseases [24]. Clinical data showed that peer-support intervention can significantly reduce diabetes distress in type 2 diabetes and AIDS orphans [25, 26]. The implementation of peer-support nursing intervention requires nurses to communicate patiently with nonsmoking patients and try to ease the nonsmoking patients' bad emotions, so as to guide them to form a scientific and healthy lifestyle.

In summary, the peers have sufficient professional knowledge and skills, which can effectively guide and manage the COPD nonsmoking patients. The psychological comfort and encouragement to nonsmoking patients cannot be replaced by medical staff. Nursing intervention based on peer support has highly clinical significance for improving the quality of life of COPD nonsmoking patients. This study did not discuss the role of family cooperation in nursing, and further follow-up studies should be carried out on the basis of formulating relevant intervention programs.

## Figures and Tables

**Table 1:** The general information of the two groups.

Group	Gender		Age	Course of disease		
	Male	Female		2–5 years	5–10 years	More than 10 years
Observation group (*n* = 50)	28	22	45.36 ± 15.68	6	24	20
Control group (*n* = 50)	27	23	46.25 ± 14.65	5	23	22

*χ* ^2^/*t* value	0.201		0.293	0.207		
*P* value	0.841		0.770	0.901		

**Table 2:** The social support scores between the two groups before and after intervention.

Time	Group	Subjective support	Objective support	Utilization of support	Total score
Before intervention	Observation group (*n* = 50)	14.96 ± 2.51	7.06 ± 1.08	7.35 ± 1.65	29.68 ± 3.68
	Control group (*n* = 50)	15.87 ± 2.41	7.21 ± 1.05	7.26 ± 1.56	29.66 ± 3.54
	*t* value	1.849	0.704	0.280	0.027
	*P* value	0.067	0.483	0.780	0.978

At 3 months after intervention	Observation group (*n* = 50)	19.65 ± 3.52	7.23 ± 1.36	8.06 ± 1.98	38.96 ± 5.23
	Control group (*n* = 50)	15.34 ± 2.36	7.26 ± 1.25	7.01 ± 1.87	30.36 ± 4.35
	*t* value	7.191	0.114	2.726	8.939
	*P* value	<0.001	0.908	0.008	<0.001

**Table 3:** The self-management efficacy scores between the two groups before and after intervention.

Time	Group	Positive attitude	Stress reduction	Self-decision	Total score
Before intervention	Observation group (*n* = 50)	29.86 ± 4.26	18.32 ± 4.23	5.36 ± 2.56	52.69 ± 9.36
	Control group (*n* = 50)	29.64 ± 4.12	18.16 ± 3.91	5.24 ± 2.49	52.74 ± 10.21
	*t* value	0.263	0.196	0.238	0.026
	*P* value	0.794	0.845	0.813	0.978

At 3 months after intervention	Observation group (*n* = 50)	36.98 ± 5.36	25.65 ± 3.56	7.65 ± 3.06	65.91 ± 11.39
	Control group (*n* = 50)	30.23 ± 4.52	18.65 ± 5.21	5.61 ± 2.87	56.36 ± 10.36
	*t* value	6.807	7.844	3.438	4.386
	*P* value	<0.001	<0.001	<0.001	<0.001

**Table 4:** The healthy lifestyle scores between the two groups before and after intervention.

Time	Group	Health responsibility	Self-actualization	Interpersonal support	Stress management
Before intervention	Observation group (*n* = 50)	13.65 ± 6.54	23.69 ± 8.21	13.68 ± 6.12	13.68 ± 3.56
	Control group (*n* = 50)	12.98 ± 6.21	23.28 ± 8.11	13.61 ± 6.36	13.21 ± 3.61
	*t* value	0.525	0.251	0.056	0.514
	*P* value	0.6016	0.802	0.955	0.656

At 3 months after intervention	Observation group (*n* = 50)	16.52 ± 6.26	26.38 ± 10.36	16.98 ± 4.52	16.89 ± 4.65
	Control group (*n* = 50)	13.84 ± 6.03	22.36 ± 4.74	13.97 ± 4.36	14.68 ± 4.06
	*t* value	2.180	2.495	3.389	2.532
	*P* value	0.032	0.014	0.001	0.013

**Table 5:** The six-minute walking distance between the two groups before and after intervention.

Group	Before intervention	At 3 months after intervention
Observation group (*n* = 50)	362.31 ± 91.24	423.67 ± 102.32
Control group (*n* = 50)	364.57 ± 93.66	382.28 ± 84.95
*t* value	0.122	2.201
*P* value	0.903	0.030

## Data Availability

The data used to support the findings of this study are included within the article.
